# Usefulness of pedagogical design features of a digital educational resource into nursing home placement: a qualitative study of nurse educators’ experiences

**DOI:** 10.1186/s12912-024-01776-5

**Published:** 2024-02-21

**Authors:** Monika Ravik, Kristin Laugaland, Kristin Akerjordet, Ingunn Aase, Marianne Thorsen Gonzalez

**Affiliations:** 1 Department of Nursing and Health Sciences, Faculty of Health and Social Sciences, University of South-Eastern Norway, Post-box 235, 3603 Kongsberg, Norway; 2 SHARE—Centre for Resilience in Healthcare, Faculty of Health Sciences, University of Stavanger, 4036 Stavanger, Norway; 3 School of Psychology, Faculty of the Arts, Social Sciences & Humanities, University of Wollongong, Wollongong, NSW Australia

**Keywords:** Digital educational resource, Nurse educator, Nursing education, Nursing home placement, Pedagogical design features, Placement learning

## Abstract

**Background:**

The rapid advancement of technology-enhanced learning opportunities has resulted in requests of applying improved pedagogical design features of digital educational resources into nursing education. *Digital educational resources* refers to technology-mediated learning approaches. Efficient integration of digital educational resources into nursing education, and particularly into clinical placement, creates considerable challenges. The successful use of digital educational resources requires thoughtful integration of technological and pedagogical design features. Thus, we have designed and developed a digital educational resource, digiQUALinPRAX, by emphasizing pedagogical design features. The nurse educators’ experiences of the usefulness of this digital educational resource is vital for securing improved quality in placement studies.

**Aim:**

To obtain an in-depth understanding of the usefulness of the pedagogical design features of a digital educational resource, digiQUALinPRAX, in supporting nurse educators’ educational role in nursing home placements in the first year of nursing education.

**Methods:**

An explorative and descriptive qualitative research design was used. Individual semi-structured interviews were conducted with six nurse educators working in first year of a Bachelor’s of Nursing programme after using the digital educational resource, digiQUALinPRAX, during an eight-week clinical placement period in nursing homes in April 2022.

**Results:**

Two main categories were identified: (1) supporting supervision and assessment of student nurses and (2) supporting interactions and partnerships between stakeholders.

**Conclusion:**

The pedagogical design features of the digiQUALinPRAX resource provided nurse educators with valuable pedagogical knowledge in terms of supervision and assessment of student nurses, as well as simplified and supported interaction and partnership between stakeholders.

**Supplementary Information:**

The online version contains supplementary material available at 10.1186/s12912-024-01776-5.

## Contributions of the paper

### What is already known

- The educational role in clinical placement education poses substantial challenges for nurse educators, such as tailoring pedagogical approaches to the learning needs and abilities of individual students.
- Digital educational resources are increasingly used in clinical placement education in nursing to enhance student learning.
- To improve the quality of clinical placement learning for student nurses, attention should be paid to the design, development, and use of digital educational resources.

### What this paper adds

- This paper adds that nurse educators experienced that pedagogical design features of a digital educational resource, digiQUALinPRAX, provided them with valuable knowledge in supervising and assessing student nurses in clinical placement education in nursing homes.
- This paper further adds that nurse educators experienced that the pedagogical design features of the digital educational resource, digiQUALinPRAX, were supportive and enhanced their role by providing possibilities for interaction and partnership between stakeholders in nursing home placement.

## Background

Nursing homes hold great potential as clinical learning arenas for first year student nurses; thus, improved quality in these clinical placement studies is crucial [1]. To provide optimal and high-quality clinical placement education and benefit from the nursing home learning potential, nurse educators play a key role in their supervision and assessment approaches [2]. Thus, nurse educators’ pedagogical approaches during clinical placement education entails meeting different levels of students’ individual learning needs and preparedness for learning [3]. From this perspective, nurse educators’ competence, engagement, pedagogical practice and experience might motivate or demotivate student nurses early on in their education, both directly and indirectly, for their future careers working with elderly in the nursing home context [4]. However, in nursing homes as an important learning context, recruiting registered nurses filling roles as students’ clinical supervisors is often a challenge [5, 6].

Nonetheless, supervising student nurses during placement education in nursing homes is reported to be a low priority among nurse educators [5]. Additionally, nurse educators in nursing homes frequently lack the formal preparation to fulfil their educational role at the expected educational level [5, 7], and are often hired to act as nurse educators for a short time during placement education [5]. Consequently, part-time nurse-educators will lead to a lack of continuity in student follow-ups [5]. Thus, addressing improved quality in clinical supervision and assessment in the Bachelor’s of Nursing Education Programs is vital [8, 9].

Tailoring pedagogical approaches to students’ individual learning needs pose substantial challenges for nurse educators [10]. Thus, supporting and enhancing nurse educators’ proficiency in supervising student nurses during placement education in nursing homes for pedagogical purposes has been suggested; this should be done using digital educational resources [8]. The present study responds to this request.

The use of digital educational resources has been increasingly developed owing to the extensively available and easily accessible internet connection [11]. These resources could be electronic (e-learning), mobile (m-learning), and online and game-based learning [12–15]. Digital educational resources are innovative educational approaches to provide knowledge in an interactive and flexible environment, thus facilitating personalised learning and improved understanding [16, 17]. Digital educational resources aimed at ensuring that student nurses have appropriate learning opportunities and that experiences are increasingly being used [18–22].

However, integrating digital educational resources in various educational institutions goes beyond easy and flexible access to these learning resources. Koehler and Mishra [23] underline the need to effectively utilise these resources for educational purposes. Thus, there is a need for educators to improve their understanding of using digital educational resources when teaching, supervising, and assessing to optimally enhance students’ learning experiences [23, 24]. Nurse educators are often underconfident and unable to optimally use digital resources, and thus are unable to understand how to modify their pedagogical approaches digitally [10, 16, 17, 25]. A recent review has reported that digital educational resources in nursing education often lacks anchoring in pedagogical theories [26]. Consequently, this will directly affect the quality of education provided to student nurses [16]. To compensate for the above mentioned shortcomings, we designed and developed a digital educational resource, digiQUALinPRAX. This resource aims to support nurse educators in developing suitable and theoretically anchored pedagogical knowledge that is adapted to student nurses during nursing home placement [8]. The co-creative process informed the educational content, design, and functionality of the digiQUALinPRAX resource, which were informed by and grounded in learning theory and principles, in line with Koehler and Mishra’s [23] ‘Technological pedagogical and content knowledge’ framework. Technological knowledge refers to knowledge of the technological characteristics, whereas pedagogical knowledge refers to how students learn best, and content knowledge refers to the domain-specific subject matter that is being taught and learned [23]. Koehler and Mishra [23] emphasise the necessity of interrelatedness and dynamic interplay between content and pedagogical and technological knowledge to effectively cater to students’ learning needs. Here, technological pedagogical knowledge refers to knowledge about the use of technology to optimally implement pedagogical approaches (i.e. the use of digital educational resources as a vehicle for the learning outcomes and experiences desired by an educator) [23].

This study aimed to obtain an in-depth understanding of how nurse educators experienced the usefulness of the pedagogical design features of the digiQUALinPRAX resource to support their role in nursing home placements. Experiences enables the identification and addressing of any issues that require improvement before the final version of a digital educational resource is released, resulting in a better pedagogical experience for nurse educators [27]. When exploring experiences about digital educational resources, experiencing educators’ feedback is crucial. This is because they have the pedagogical competence and experience necessary to create resources that align with curriculum goals [28].

## Methods

### Design

The current study applied an explorative and descriptive qualitative research design. This is appropriate for investigating an unexplored subject descriptively, along with its characteristics [29]. The study is part of a larger research project [8] that developed the digiQUALinPRAX resource. The digiQUALinPRAX resource was co-created with key stakeholders (i.e. student nurses, nurse educators, registered nurse mentors, e-learning designers and researchers) to enhance quality in nursing home placements, including the support and enhancement of the nurse educators’ role. For a detailed description of the overall co-creative development process, see Laugaland et al. [30].

### Educational placement context

In Norway, becoming a registered nurse requires the successful completion of a 3-year Bachelor’s curriculum programme (180 credits), developed in accordance with the European Directive [31] and national regulations [32]. Half of this nursing education programme in Norway and elsewhere in Europe comprises of the clinical placement component [31, 32]. As part of their professional responsibilities, the qualified and experienced registered nurses fulfilled the role of registered nurse mentors for students during their clinical placements. They focused on mentorship rather than actively teaching and developing the students’ competencies, indicating that mentoring by registered nurses was service-led rather than educationally driven. Although these registered nurse mentors possessed appropriate qualifications, they lacked formal academic educator competencies. Meanwhile, nurse educators bridged the gap between academic and placement knowledge. They possessed pedagogical knowledge and played a vital role in supporting, supervising and assessing student nurses. Nurse educators, who hail from the academic setting, bear the pivotal responsibility for the final decision of whether students pass or fail. They support, supervise and assist students and their registered nurse mentors during clinical placement and take care of the collaboration between these two stakeholders. The clinical experience for student nurses was set up through a collaborative effort between nurse educators from the university setting and registered nurse mentors in the clinical setting. In this collaboration, nurse educators were crucial to facilitating clinical learning experiences by securing optimal learning situations in the nursing homes in line with the educational learning outcomes. In these learning situations, the registered nurse mentors served as facilitators, mentors and role models. They also consistently provided valuable insight from their professional experiences, offered daily mentoring, and delivered feedback. This collaboration between the stakeholders aimed to help students in bridging the gap between the knowledge gained in the university setting and their clinical experiences in the nursing homes.

### The digiQUALinPRAX resource being experienced in the study

**Fig. 1:**
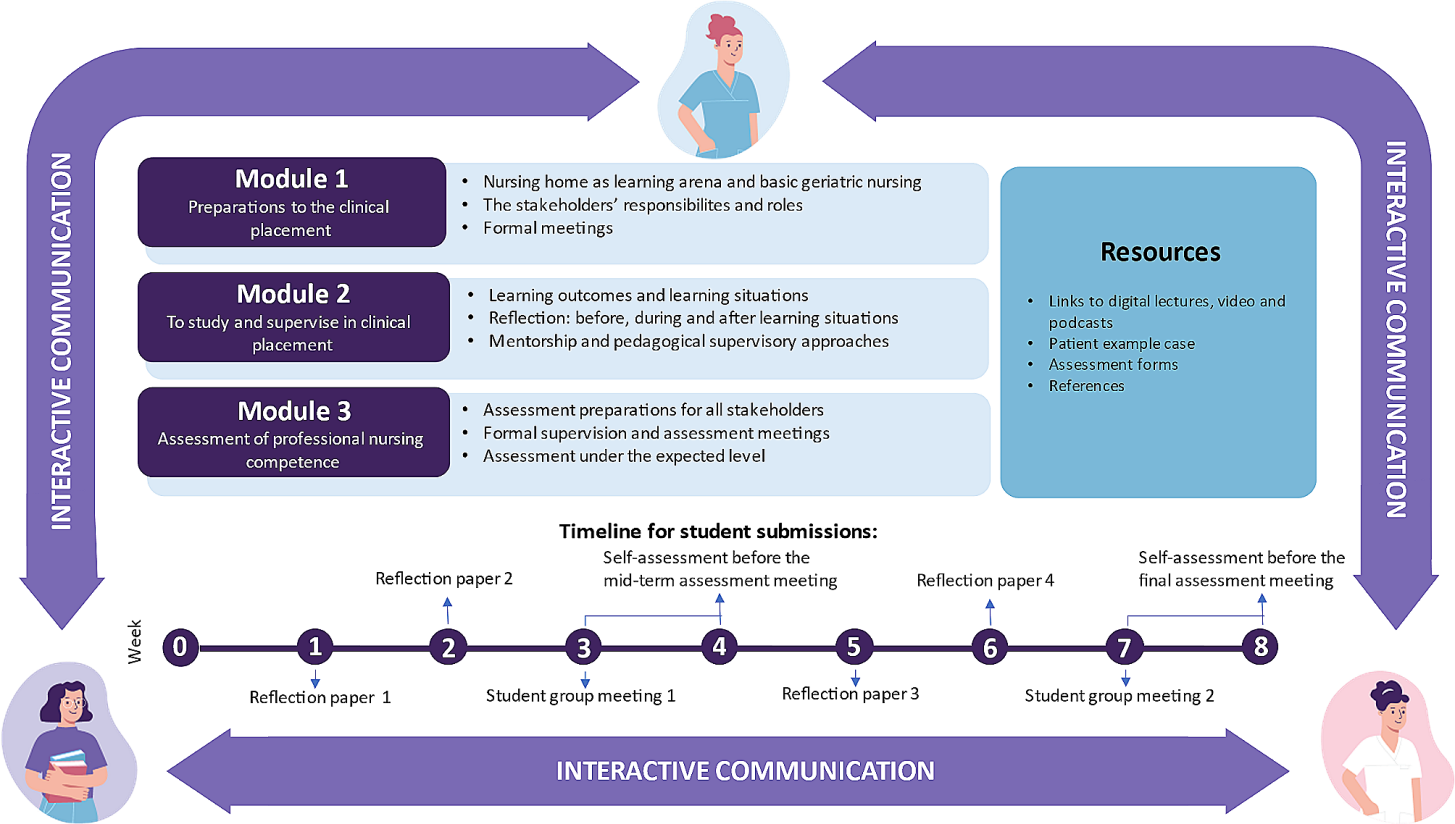
Core components of the digiQUALinPRAX resource

The digiQUALinPRAX resource (Fig. 1) is a password-protected learning management system named Canvas (website), a technology that is used to plan, implement, and assess learning processes [33]. The overall educational aim of the digiQUALinPRAX resource was to enhance quality in nursing home placements by addressing students’ learning and the mentorship practices of educators and registered nurses (i.e. supervision and assessment). Nurse educators and registered nurse mentors, in turn, utilised this resource to enhance their teaching strategies, coordinate clinical placement activities, and ensure meaningful and enriching learning experiences for their students. The digiQUALinPRAX resource was designed to support the collaborative efforts of the stakeholders, fostering a dynamic and effective learning environment within the entire context of clinical placement education in nursing homes.

The digiQUALinPRAX resource consists of several core components as design features (i.e. *interactive components, content components*, and *resource components*). The *interactive components* entailed features such as file sharing and messaging (through a dialogue forum), enabling stakeholders to interact with each other during the placement period. The dialogue forum provided a digital room where nurse educators and registered nurse mentors could provide written feedback on students’ assignments submitted through the digital educational resource.

Furthermore, the *content components* of the digiQUALinPRAX resource consisted of three content modules, including practical, educational, and contextual knowledge relevant for clinical placement in nursing homes. The three content modules were organized with different topics by the following titles: (1) Preparation to the clinical placement; (2) To study and supervise in clinical placementt; and (3) assessment of professional nursing competence. The first content module contained literature on pre-placement information, addressing the nursing home as a learning arena, role expectations, and schedule of the placement period. This content module further included a fixed time structure with predefined meetings. Additionally, an overview of the students’ theoretical educational content before placement and thus, their expected level of professional competence, was provided. The second content module contained literature on how to study, learn, and provide appropriate mentoring. This module provided examples of learning situations, as well as a description of students’ competence domains. These were tailored to accommodate the students’ learning objectives and mentoring activities. The use of reflection as a learning strategy was emphasized in this module. During the eight-week placement period, students had to write several reflection papers about various topics. The module further facilitated possibilities for nurse educators to provide written feedback on the reflection papers to stimulate and enhance students’ reflection skills. The third content module focused on assessment practices and provided information about formal and formative assessments. This was done by thoroughly describing the assessment forms through exemplifying how they could be used based on one specific patient situation. The formal assessment documents were all available directly in the digiQUALinPRAX resource.

The *resource components* of the digiQUALinPRAX resource consisted of practical, educational, and context-specific resources. These resources were illustrations, podcasts, video lectures, reflective activities, case-related activities, and resources to support nurse educators’ educational roles. Additional resources were study requirements, advice, and summaries of the core components.

### Study sample and recruitment

The target group for this study was nurse educators who were employees at one university in Norway, at which the digiQUALinPRAX was explored. The inclusion criterion was nurse educators having used the digiQUALinPRAX resource during an eight-week clinical placement period in nursing homes.

A purposive sampling strategy [34] was applied to recruit participants. After obtaining approval from the Vice-Dean of the Faculty of Health Sciences, potential nurse educators were sent recruitment e-mails. The e-mails contained general study information and a waiver of consent. Invitations were sent openly to nurse educators who had a supervisory responsibility to student nurses in nursing home placement. Six nurse educators consented to participate and received complete verbal information about the study. We considered these six nurse educators to be a representative sample [35] because they had used the digiQUALinPRAX resource during an eight-week placement period.

### Research context

One week before the clinical placement period, the digiQUALinPRAX resource was precented and made accessible to the stakeholders (i.e. nurse educators, student nurses and registered nurse mentors) involved in the overall study. All stakeholders had access to the digiQUALinPRAX resource throughout the eight-week clinical placement period. As the target group in this study, the nurse educators were the only stakeholders possessing pedagogical knowledge and thus played a vital role in supporting, supervising, and assessing student nurses during their placements using the digiQUALinPRAX resource. Furthermore, they were responsible for collaborating with registered nurse mentors in their supervision of student nurses and in the use of the digiQUALinPRAX resource.

### Data collection

Individual qualitative interviews with the six nurse educators were conducted for data collection. Data from individual interviews are valuable when the insight and understanding of participants’ perceptions, experiences, thoughts, and suggestions with respect to a given subject are of interest [29]. The qualitative nature of our research design, employing an in-depth exploration of the experiences of nurse educators, warranted a focus on detailed and context-specific insight rather than a large sample size [35]. The selected sample size was determined through a careful balance between power of information and the specific group of nurse educators with unique experience characteristics, which contribute to the depth of the analysis and results [35]. The nurse educators’ interviews were arranged in an academic nursing setting immediately after the eight-week clinical placement period in nursing homes for first year student nurses. Data were collected by the first author in April 2022.

A semi-structured interview guide was employed, addressing themes such as supervision and assessment possibilities, partnership, interaction and communication opportunities, and knowledge provided by the digital educational resource (see Supplementary File 1). Participants were offered opportunities to speak freely about their experiences, with follow-up questions where appropriate. Owing to COVID-19 restrictions, all interviews were conducted through a virtual platform via ZOOM using video and sound. This interview format encouraged two-way communication, allowing for conversations on relevant themes [29]. The interviews were audio recorded and lasted between 56 and 99 min. The six nurse educators provided rich information on their experienced usefulness of the pedagogical design features of the digiQUALinPRAX resource. The more information the participants held relevant to the actual study, the lower the number of participants needed [35].

### Data analysis

All audio files were transcribed verbatim, resulting in text describing spoken words from the audio files underpinning the analysis, as recommended by Halcomb and Davidson [36]. After transcription of the audio files, text data were analysed using systematic text condensation in line with Malterud [37] (e.g. an explorative and descriptive method for thematic analysis that addresses the characteristics and essence of the subject being studied). NVivo software [38] version 12 was used for data analysis.

Data analysis was inductive; the text was re-read for a general overview and to familiarise the researchers with the content. Preliminary themes were captured in the first phase of the analysis. In the second phase, meaning units were identified and organised in relation to each of the themes captured in phase one. This data extraction approach entailed the decontextualization of the text: to be separated into parts or segments and removed from the belonging context [37]. Each meaning unit was coded and sorted into code groups. These were created in relation to each theme and provided a platform for the next phase of the analysis, in which a deeper meaning of experience was sought. In the third phase, the code groups were divided into sub-groups; the meaning units in the sub-groups were rewritten into condensates [37]. I–form was chosen to optimally represent the participants’ views, and their own words were used to maintain the original terminology. After completing the condensates, illustrative quotations (translated into English) were selected. Adjustments were made to provide a clearer understanding of the statements. In the fourth phase, the decontextualised text was recontextualised and synthesised; that is, parts were put into a new context while being true to the text from which the data were extracted. The condensed text from each sub-group within the code groups ‘went beyond’ the condensates, and new interpretive descriptions about the subject being studied were generated, to be presented in a third-person format [37]. Throughout the analysis, the first and last authors discussed the codes, sub-categories, and categories until reaching consensus. The recontextualisation resulted in two categories and five sub-categories.

### Rigour

To ensure the trustworthiness of this qualitative study, credibility, dependability, transferability, and confirmability were considered 

[39]. Credibility was ensured in this study using an interview guide to establish consistency in the data collection process. Furthermore, video recordings (ZOOM) and transcription of the interviews verbatim helped ensure an accurate and complete representation of the nurse educators’ responses. Dependability was ensured by describing data collection and analysis in detail. NVivo was used to organise and visualise the data. Moreover, nurse educators’ arguments were quoted to show the links between the findings and data. To enhance the transferability, detailed descriptions of the research process were provided. Investigator triangulation was applied, where the first and last authors engaged in discussions and revisited the transcripts to ensure that the interpretations were supported by the data transcripts. The first and last authors held regular meetings to discuss the data analysis and ensure confirmability. Nurse educators were selected to provide in-depth data. Few participants were needed; information power was attained owing to the sample specificity and quality of dialogue [35].

### Ethical considerations

This study was approved by the Norwegian Centre for Research Data (2018/61,309 and 489,776) and the university included prior to data collection. According to national regulations, approval from a medical ethical committee (Regional Committees for Medical and Health Research Ethics) to collect this type of data was not necessary. The study was conducted in accordance with the ethical principles of the Declaration of Helsinki [40]. The consolidated criteria for reporting qualitative research (COREQ) guideline was used to report the study. All participants received written and verbal information about the study, including the voluntary nature of participation and the right to withdraw from the study. All participants provided written informed consent, while non-participants refused to take part in the study. To ensure confidentiality, participants’ characteristics such as age, sex, educational background, and years of experience in placement education supervision were not provided. All data were anonymised and securely stored to ensure confidentiality and protect private information.

## Results

The qualitative analysis of nurse educators’ experiences in relation to the pedagogical design features of the developed digiQUALinPRAX resource resulted in perceptions and reflections of the following key categories: (1) supporting supervision and assessment of student nurses and (2) supporting interaction and partnership between stakeholders (Table 1).

**Table 1:** Categorisation of educators’ perceptions about the digital educational resource

Categories	Sub-categories
SUPPORTING SUPERVISION AND ASSESSMENT OF STUDENT NURSES	• Offering possibilities to provide students feedback on their written assignments (directly in the dialogue forum) • Offering possibilities to encourage students’ reflections • Facilitating provision of summative assessments of students’ study progression
SUPPORTING INTERACTION AND PARTNERSHIP BETWEEN STAKEHOLDERS	• Simplifying and supporting interactions and cooperation between the stakeholders • Simplifying and stimulating communication using the dialogue forum

### Supporting supervision and assessment of student nurses

Nurse educators experienced that the pedagogical design features of the digiQUALinPRAX resource allowed for supporting supervision and assessment in terms of giving students feedback on their written assignments, encouraging their reflections and facilitating summative assessments of their study progression.

#### Offering possibilities to provide students feedback on their written assignments (directly in the dialogue forum)

Nurse educators positively experienced the interactive component of the digiQUALinPRAX resource as providing possibilities for giving written feedback on students’ written submissions in the dialogue forum, both from nurse educators and registered nurse mentors. Moreover, nurse educators experienced the digiQUALinPRAX resource to be useful as it forced them to provide feedback on all the students’ written assignments. However, they found it challenging to provide written feedback via the dialogue forum because it was not possible to simultaneously review students’ submissions while providing written feedback through the digiQUALinPRAX resource.

*I had to go in and out of the dialogue forum when written feedback was provided on students’ submissions to read the text of the submissions. (Informant 4)*

#### Offering possibilities to encourage students’ reflections

Nurse educators experienced the digiQUALinPRAX resource to be useful in terms of guiding specific topics for the reflection papers that were written and submitted by student nurses during placement, stating that it contributed to a more common focus on student learning during placement. They also positively experienced that the digiQUALinPRAX resource ensured that the topics of the reflection papers were not arbitrary and dependent on the individual nurse educators’ personal recommendations and preferences, which helped them provide more consistent supervision with a focus on student learning.

*Learning focus became more common for student nurses because the digital educational resource guided them in terms of the topics that they should write about. (Informant 5)*

Furthermore, nurse educators experienced the digiQUALinPRAX resource-led reflection papers written by students as a useful source of information. Specifically, these papers allowed nurse educators to gain valuable insights into each student’s learning and knowledge levels, enabling them to identify areas requiring further attention for learning and development. They experienced such papers as providing them with a clear understanding of the aspects that they should focus on and providing feedback to students. This approach enabled them to help correct misunderstandings and fill gaps in students’ academic and professional knowledge.

The use of the digiQUALinPRAX resource was experienced by nurse educators to help both inexperienced and experienced nurse educators when supervising students in nursing home placements and guiding reflection group meetings. Inexperienced nurse educators were helped to understand the concept and purpose of reflection and how to encourage students to engage in reflective processes. They further faced experienced educators as helping them obtain a better structure for the reflection group meeting, focusing on the reflection group towards the real education levels and learning outcomes. Nurse educators experienced that the use of the digiQUALinPRAX resource in reflection group meetings resulted in a superior focus on students’ learning processes. Moreover, supervision became more student- rather than teacher-centred.

*I became more like a facilitator than a nurse educator in the reflection group meetings because the digital educational resource-led questions helped me encourage the students to reflect amongst themselves and with me as an educator. (Informant 1)*

Nurse educators experienced the digiQUALinPRAX resource-led pedagogical materials as useful in influencing students’ engagement and verbal activity during reflection group meetings.

*The case is great to work on together with the students. Additionally, the students enjoyed working on the case, they became actively engaged. (Informant 4)*

Nurse educators experienced that digiQUALinPRAX resource-led pedagogical materials, such as cases, care plans, and reflection questions, served as a foundation for the reflection group meetings and consequently, facilitated students’ development of professional understanding and competence about the nursing profession.

#### Facilitating provision of summative assessments of students’ study progression

Several nurse educators experienced that using digiQUALinPRAX resource-led single patient situations as the basis for providing summative student assessments restricted the ability to comprehensively assess student progression on all items of the assessment form.

*Sufficient data were unavailable to provide summative assessments of student progression using only one patient situation. (Informant 2)*

Some nurse educators included multiple patient situations as the basis for providing summative student assessments, even though this was not guided by the digiQUALinPRAX resource; they experienced this to be beneficial for ensuring comprehensive coverage of all items on the assessment form. The nurse educators felt that this allowed students to demonstrate their study progress and identify areas of improvement.

*It was unproblematic that the varied assessment items were written based on different patient situations because they provided more information about the student’s progression. (Informant 1)*

*Students completed the assessment based on several patient situations to show their knowledge well enough. (Informant 6)*

Nurse educators experienced their role in summative assessment meetings as more constructive when registered nurse mentors completed the digiQUALinPRAX resource-led assessment form prior to the summative assessment meetings. This was because they adopted a cautious approach during assessment meetings as the registered nurse mentors’ verbal participation increased when they filled in their digiQUALinPRAX resource-led student evaluation form prior to the assessment. Specifically, nurse educators regarded it as a positive experience when registered nurse mentors provided clear verbal feedback on areas where students required further progress during the placement study.

*Registered nurse mentors’ threshold for being verbally engaged during summative assessment situations was lowered because they had completed the digital-educational resource-led assessment form prior to the assessment meetings (Informant 1)*.

*Registered nurse mentors who had prepared themselves by writing in the digital educational resource-led assessment form were more verbally engaged during the summative assessment meetings. (Informant 6)*

The registered nurse mentors’ clear and precise communication of students’ areas that required improvement during the placement was experienced positively by the nurse educators, as it provided them with a clear focus on what to prioritise when further supervising the students’ progress.

*When the registered nurse mentor completed the digital-educational resource-led assessment form and was verbally engaged during the summative assessment meeting, the student’s next steps became clear. (Informant 2)*

### Supporting interaction and partnership between stakeholders

Nurse educators experienced that the interactive digiQUALinPRAX resource design contributed to increased support for interactions and establishing partnerships between stakeholders through stimulating communication and cooperation between stakeholders.

#### Simplifying and supporting interactions and cooperation between the stakeholders

Nurse educators experienced that the digiQUALinPRAX resource-led timeline enabled them to schedule equal in-person supervision group meetings with students during their clinical placement. Further, they experienced interactions and cooperation with students as important for encouraging students to engage in appropriate and meaningful learning processes and as a feature of conducting accurate student assessments during placements.

*I established closer contact with the students because I used the digital education resource. (Informant 6)*

Additionally, nurse educators experienced their cooperation with registered nurse mentors to have improved because of the use of the digiQUALinPRAX resource; that is, the registered nurse mentors contacted nurse educators more during clinical placement compared with before. As part of the appropriate student supervision, nurse educators emphasised the importance of a proper relationship between the clinical placement setting and various registered nurse mentors.

*The threshold for the registered nurse mentors to contact me as an educator was lowered owing to the use of the digital educational resource. (Informant 1)*

#### Simplifying and stimulating communication using the dialogue forum

Nurse educators experienced the dialogue forum usage to be unclear, and gave feedback on how they could appropriately use the dialogue forum (i.e. the digiQUALinPRAX resource-led interactive component that facilitates communication between the stakeholders during the placement).

*Clarifications about the use of the dialogue forum should have been made because we were not used to making discussions in this forum. (Informant 1)*

However, the nurse educators considered that the dialogue forum should only provide possibilities for communication between the varied stakeholders included in the supervision collaboration: the students, registered nurse mentors, and nurse educators. Nurse educators experienced this as a necessity for a dialogue forum that also fosters transparency and open communication between the nurse educator and their student group, such as an information channel providing possibilities for a nurse educator to disseminate the same information to all students in the student group simultaneously.

*It is out of question sending information to students individually that can be disseminated to all students. (Informant 5)*

Nurse educators experienced it that it was necessary for a dialogue forum to provide possibilities for confidentiality (e.g. as an alternative to emails for stakeholder communication). Moreover, confidentiality was not ensured in cases where students might not pass their placement. Students’ exclusion from the forum was requested when discussions solely between the educator and registered nurse mentor might be necessary.

*I cannot raise challenging student situations in a dialogue forum if the student has access to the digital room. (Informant 3)*

## Discussion

The current study aimed to explore and describe how nurse educators experienced the usefulness of the pedagogical design features of the digiQUALinPRAX resource from their perspective. Nurse educators’ positive experiences regarding the digital educational resource highlighted the pedagogical design features as unique features, improving their supervision and assessment of student nurses during clinical placement education in nursing homes. These findings align with those of previous studies suggesting that pedagogical design is essential for creating digital educational resources [26]. This is an important finding, as pedagogical design features are often overlooked in the technologies designed to enhance and support clinical placement education in Bachelor’s nursing programmes [26, 41].

Nurse educators experienced the interactive communication features of the digiQUALinPRAX resource as a valued component, as it enhanced their ability to provide written feedback on students’ submissions for their learning processes. This finding is also important, as earlier research [42, 43] has revealed that many students received insufficient written feedback on their submissions from nurse educators during clinical placement education. This inappropriate feedback may have a negative influence on students’ learning experiences, whereas it might hinder them in their ability to identify areas in which they need to improve and further study to close their gap in knowledge [43]. Hence, feedback plays a crucial role in supporting students in understanding their strengths and weaknesses, thereby helping them achieve their learning outcomes [44–46].

Providing written feedback on student submissions was also important for nurse educators in our study. File sharing, as an interactive part of the digiQUALinPRAX resource, enabled the nurse educators to gain insights into the students’ knowledge levels and provide feedback based on their individual learning needs. This aligns with the sociocultural learning perspective, which underscores Vygotsky’s [46] theory of learning and development. According to this theory, interactions with more proficient persons can help the learner advance to the next level of knowledge and understanding within their zone of proximal development.

Our findings also revealed that nurse educators experienced that scheduled digiQUALinPRAX resource-led submissions contributed to students receiving frequent feedback. This finding is in line with the results of Bosse et al. [47], who emphasised the benefits of receiving frequent feedback, as it led to better learning outcomes. Moreover, this illustrates that considering integrating pedagogical design features when developing digital educational resources is valuable in stimulating nurse educators to facilitate students’ learning processes.

Nurse educators noted that pedagogical design features of the digiQUALinPRAX resource helped them encourage students to actively engage in reflective thinking, both verbally and in writing. Reflective thinking involves critically analysing experiences, considering one’s thoughts and emotions and examining the broader context [48]. Improved learning through reflective-thinking processes among students has also been considered in prior research, showing that it can deepen the comprehension of learning objectives and increase the awareness of decision-making in clinical reasoning [49, 50]. This indicates the importance of possessing reflective thinking skills, not only in improving self-directed learning but also in delivering high-quality patient care [48, 51].

The study findings indicated that pedagogical design features of the digiQUALinPRAX resource facilitated a shift in the nurse educators’ role in reflection group meetings. The shift entailed moving from being nurse educators who often communicated their knowledge to assuming the role of facilitators who guided discussions and encouraged students’ reflections and critical thinking. This pedagogical approach prioritises a student-centred learning model, enabling student nurses to construct their understanding actively rather than passively receiving the presented information [49, 50]. This finding is important because nurse educators often fail to involve students in reflective-thinking activities during their educational process, resulting in a lack of student participation and difficulties in comprehending learning objectives [6, 48, 52–55]. Regarding this issue, Dalsmo et al. [52] revealed that nurse educators were often ‘invisible’ in students’ learning processes during nursing home placement, hindering students’ ability to participate fully and comprehend the learning objectives.

Nurse educators positively experienced that pedagogical design features of the digiQUALinPRAX resource encouraged registered nurse mentors to provide a written assessment concerning both the strengths and weaknesses of student progression prior to summative assessment meetings, resulting in registered nurse mentors becoming more verbal during the meetings. Several studies have reported that nurse educators experience challenges in assessment meetings because of registered nurse mentors’ silence [42, 52, 56]. When nurse educators in our study experienced that registered nurse mentors wrote and verbalised what was expected from the students to work on during the remaining placement study, they were given opportunities to gear their student supervision towards the learning needs to focus on. From this perspective, nurse educators experienced that pedagogical design features of the digiQUALinPRAX resource facilitated both themselves and the registered nurse mentor to develop a common understanding regarding students’ learning needs. Previous research has revealed that educators and registered nurse mentors often have different expectations regarding students’ learning needs during placement studies [56, 57]; thus, creating a common understanding among the stakeholders is crucial for effective student supervision.

Having a clear structure in the form of a timeline was a distinct pedagogical design feature of the digiQUALinPRAXresource that enhanced nurse educators’ student supervision abilities. They reported that the timeline specifying the number of physical meetings to be held during the placement period (and when they occurred) contributed to nurse educators being able to organise physical meeting frequency more equally. This is a valuable pedagogical design feature of the digiQUALinPRAX resource because dissatisfaction among students with their nurse educators’ physical presence in follow-ups during placement studies has been reported [3, 6]. Further, the nurse educators experienced the timeline-defined specific topics for the reflection papers positively, ensuring that the topics did not become dependent on individual nurse educators’ preferences. In Ravik et al. [42], nurse educators requested greater consensus among themselves to enhance student supervision. Differences among nurse educators might be perceived as unjust by students and could account for some students learning more than others during placement studies because they receive more personalised attention from their nurse educators [58]. Therefore, including timeline-defined physical meetings for nurse educators and defined topics of the reflection papers might help address this issue. Both Cant et al. [3] and Laugaland et al. [8] reported that inconsistency between educators hinders improvements in students’ learning. Moreover, it was deemed essential for nurse educators to be physically present during clinical placement to ensure that they maintained suitable communication with registered nurse mentors. These findings are consistent with those of previous studies, suggesting that appropriate relationships and communication between stakeholders are critical for creating a supportive and collaborative learning environment for students [3].

Nurse educators positively experienced the inclusion of interactive design features in the form of a dialogue forum. This forum played a vital role in facilitating interactions between students and the stakeholders involved in overseeing and supervising students during their nursing home placement. Previous research supports the notion that this interactive design feature, integrated into digital educational resources, is essential for effectively implementing and utilising technology to enhance student supervision [20, 42, 59]. Notably, the presence of such dialogue forums, which enables interaction among stakeholders, has been reported as an indicator of satisfaction with digital educational resources [20]. This underscores the importance of fostering a sense of belonging within a learning community, which has been recognised as vital to student nurses’ placement learning experiences [60]. Even though the nurse educators highlighted the importance of a dialogue forum contributing to openness between all stakeholders during student supervision, they pointed out that the dialogue forum should be available for the nurse educator and registered nurse mentor only, allowing for confidential dialogues in challenging situations. Therefore, the interactive dialogue forum can create an atmosphere where nurse educators and registered nurse mentors can share concerns, exchange perspectives, and collaboratively develop strategies to address the challenges faced by students [42]. This is in line with previous research suggesting that open and confidential communication among stakeholders contributes to finding common ground and fostering productive resolutions [57].

### Limitations and future research directions

Some limitations should be considered when interpreting the results of this study. Individual interviews were conducted by the first author, who was also involved in the design and development of the digital educational resource, digiQUALinPRAX. However, the first author was unknown to the participants, and lived and worked in another part of the country. Additionally, the participants were encouraged to frankly share their experiences and opinions regarding the use of digital educational resources. Despite the small sample size, the rich information that they provided allowed for the in-depth feedback and experiences we had aimed for in this study. It is, however, important to acknowledge that while the results may provide valuable insight into the experiences of the nurse educators, transferability to broader populations may be limited. Qualitative research is needed to explore and deepen these findings from the perspectives of student nurses and registered nurse mentors for the improvement of digiQUALinPRAX. Moreover, quantitative research is essential to providing knowledge about the effectiveness of digiQUALinPRAX in measuring and assessing student learning. Additionally, to broaden the applicability of the current study, it is recommended to explore the results across diverse healthcare educational settings, such as hospital settings for second- or third-year students. It is also suggested to explore revisions to the digital educational resource that would enable its adaptation to other internships within nursing education. This expanded exploration may contribute to the transferability of the results and enhance the broader relevance of the study’s implications.

## Conclusions

The nurse educators gave in-depth information on how they experienced the usefulness of the pedagogical design features of the digiQUALinPRAX resource, developed to support their role in nursing home placements. The digiQUALinPRAX resource was experienced to display several positive pedagogical design features for enhancing the supervision and assessment of student nurses, while also promoting possibilities for interactions and partnerships among stakeholders. Notably, its inclusion of a timeframe was experienced as beneficial for ensuring greater consistency among nurse educators in student supervision. Additionally, its resource design facilitated student feedback, enabled nurse educators to better understand students’ current knowledge levels as well as their need for further supervision and learning. Furthermore, it was experienced as positive that pedagogical design features of the digiQUALinPRAX encouraged nurse educators to engage students in the reflective-thinking processes. Moreover, it was positively experienced that pedagogical design features of the digiQUALinPRAX contributed to registered nurse mentors becoming more verbal in assessment meetings, which also positively contributed to nurse educators’ further supervision of students during nursing home placement.

### Electronic supplementary material

Below is the link to the electronic supplementary material.

Supplementary Material 1

## Data Availability

To access the data in this study, please contact the corresponding author.
