# Supplementary material for: Usefulness of pedagogical design features of a digital educational resource into nursing home placement: a qualitative study of nurse educators’ experiences
Source: BMC Nurs. 2024 Feb 21;23:135. doi: 10.1186/s12912-024-01776-5 (PMC10882782; doi:10.1186/s12912-024-01776-5)
Supplement: Supplementary file 1 — Supplementary Material 1 [file 12912_2024_1776_MOESM1_ESM.docx]

**INTERVIEW GUIDE**

| **FOCUS: Experiences from utilizing a digital educational resource** |
| --- |
| - **Now the students have completed their clinical placement, and the period for using the digital educational resource has concluded. I would appreciate hearing about your experiences with the digital educational resource.**   **1: Comprehensive relevance and practical value**   - **Kindly share your experiences with the digital educational resource throughout the clinical placement period.**   **2: Layout and structure**   - **What are your experiences regarding the layout and structure of the digital educational resource?**   **3: Content, utilization, and practical value**   - **What are your experiences in utilizing the three main components (part one, two and three) of the digital educational resource?** - **Which resources included in the digital educational resource have you experienced to be the most useful, and why? (podcast, lectures, case, reflection questions and resource for supervision, learning and assessment form – with competence areas)** - **What aspects of the digital educational resource were important enabling active use throughout the clinical placement period?**     **4: Supervision practice/improved supervision competence**   - **Do you experience that the digital educational resource influenced your supervision during clinical placement, if so, in what ways?** - **Do you experience that the digital educational resource influenced your role as a supervisor during clinical placement, if so, in what ways?** - **Assessment in clinical placement**   - *What experiences do you have with assessment practices we have included into the digital educational resource?*   - *What experiences do you have related to the self-assessment form/resource for learning, supervision, that we have included into the digital educational resource?*   **5: Collaboration during clinical placement (student/registered nurse mentor – student/nurse educator - nurse educator/registered nurse mentor - student/nurse educator/registered nurse mentor)**   - - *Did you utilize opportunities for collaboration provided by the digital educational resource – if so, why, how, and how often?*   - *Do you experience any changes regarding collaboration with registered nurse mentors and students when using the digital educational resource, if so, how have these changes manifested?*   - *Do you experience the communication with students and registered nurse mentors to be changed or become easier to initiate when using the digital educational resource – if so, in what ways?*   **Do you have anything else to add before we conclude the interview?** |
